# Deep learning-based pathway-centric approach to characterize recurrent hepatocellular carcinoma after liver transplantation

**DOI:** 10.1186/s40246-024-00624-6

**Published:** 2024-06-05

**Authors:** Jeffrey To, Soumita Ghosh, Xun Zhao, Elisa Pasini, Sandra Fischer, Gonzalo Sapisochin, Anand Ghanekar, Elmar Jaeckel, Mamatha Bhat

**Affiliations:** 1 Ajmera Transplant Centre, University Health Network, Toronto, ON Canada; 2 Division of Gastroenterology & Hepatology, University of Toronto, Toronto, ON Canada; 3 Institute of Medical Sciences, University of Toronto, Toronto, ON Canada; 4 Toronto General Hospital Research Institute, University Health Network, Toronto, ON Canada; 5 Princess Margaret Cancer Centre, University Health Network, Toronto, ON Canada; 6 Department of Medicine, University of Toronto, Toronto, ON Canada

**Keywords:** Recurrent hepatocellular carcinoma, Liver transplantation, Machine learning, PI3K/Akt signaling pathway & interleukin 6

## Abstract

**Background:**

Liver transplantation (LT) is offered as a cure for Hepatocellular carcinoma (HCC), however 15–20% develop recurrence post-transplant which tends to be aggressive. In this study, we examined the transcriptome profiles of patients with recurrent HCC to identify differentially expressed genes (DEGs), the involved pathways, biological functions, and potential gene signatures of recurrent HCC post-transplant using deep machine learning (ML) methodology.

**Materials and methods:**

We analyzed the transcriptomic profiles of primary and recurrent tumor samples from 7 pairs of patients who underwent LT. Following differential gene expression analysis, we performed pathway enrichment, gene ontology (GO) analyses and protein-protein interactions (PPIs) with top 10 hub gene networks. We also predicted the landscape of infiltrating immune cells using Cibersortx. We next develop pathway and GO term-based deep learning models leveraging primary tissue gene expression data from The Cancer Genome Atlas (TCGA) to identify gene signatures in recurrent HCC.

**Results:**

The PI3K/Akt signaling pathway and cytokine-mediated signaling pathway were particularly activated in HCC recurrence. The recurrent tumors exhibited upregulation of an immune-escape related gene, *CD274*, in the top 10 hub gene analysis. Significantly higher infiltration of monocytes and lower M1 macrophages were found in recurrent HCC tumors. Our deep learning approach identified a 20-gene signature in recurrent HCC. Amongst the 20 genes, through multiple analysis, *IL6* was found to be significantly associated with HCC recurrence.

**Conclusion:**

Our deep learning approach identified PI3K/Akt signaling as potentially regulating cytokine-mediated functions and the expression of immune escape genes, leading to alterations in the pattern of immune cell infiltration. In conclusion, *IL6* was identified to play an important role in HCC recurrence.

**Supplementary Information:**

The online version contains supplementary material available at 10.1186/s40246-024-00624-6.

## Introduction

Hepatocellular carcinoma (HCC) ranks as the third leading cause of cancer-related mortality worldwide, with a 5-year survival rate of less than 12% [1, 2]. Liver transplantation (LT) is offered as a curative treatment for HCC [3]. However, HCC has emerged as the leading indication for LT worldwide in recent years [3, 4]. HCC recurrence after LT poses a significant clinical challenge, with reported rates of 10–20% in the first year after LT [5–8]. HCC recurrence also affects the post-transplant survival of up to 21% of patients, with a median survival of 10.6 months [9].

Recent investigations into the genomic features of HCC tumor relapse made use of next-generation DNA sequencing in primary and recurrent HCC tumor samples [10, 11]. The studies have reported that *TP53*, *TERT*, *CTNNB1*, *TSC2*, and *JAK1* mutations and the HERH-4-miR-29b/c-CCNA2 axes were associated with the progression of recurrent HCC [10, 12]. However, transcriptomic studies comparing primary and recurrent HCC tumors are still limited. Our study addresses this gap by analyzing the transcriptomic clonal evolution in HCC recurrence from a rare collection of primary and recurrent tumors.

Machine learning, especially deep learning with artificial neural networks, has proven highly effective in genetics and genomics research [13]. It enables identification of complex patterns within large datasets, which may be too intricate for manual analysis [13]. Here, we present a transcriptome-wide study of HCC recurrence with primary and recurrent tumors. We further investigate the involved mechanisms, altered immune cell infiltration and through a deep learning-based approach identify gene signatures that are differentially modulated between primary and recurrent tissues. Overall, our study provides a better understanding of the genetic alterations and important gene signatures that differentiate primary from recurrent tumors.

## Methods

### Patient population

This study included patients who underwent LT for HCC between 2004 and 2016. Paired tumor samples were collected from 7 patients at the initial resection and recurrence. Patients with a maximum of 4 lesions of the primary tumor were selected, and only the largest dominant lesion was retrieved to obtain RNA from archived formalin-fixed, paraffin-embedded (FFPE) samples available for both groups. The RNeasy FFPE Kit (QIAGEN) was used to obtain RNA from the FFPE samples in accordance with the manufacturer’s instructions. The characteristics of the tumors, including grade, size, number of tumors, presence of microvascular invasion, and associated AFP, were collected for the explant or resected lesions. The date of recurrence, number of lesions identified, AFP peak, and type of therapy used for treatment were retrieved from patients with recurrent lesions. Trough level of the immunosuppressive medication from the time of transplant to the time of recurrence was also collected from the transplant patients. Patients’ clinicopathological characteristics on HCC recurrence are summarized in Table 1. The study protocol was in accordance with the Declaration of Helsinki. The University Health Network (UHN) Research Ethics Board (REB#15- 9989) approved the request for a waiver of consent to retrieve retrospective data and tissue. Research followed Helsinki and Istanbul Declarations, approved by relevant ethics committees with written consent from all subjects.

**Table 1:** Clinicopathological characteristics of patients transplanted for HCC.

Variable	Transplant patients (*n* = 7)
Mean age (range), y	64 (58–71)
Gender, No. of men (%)	7 (100%)
Etiology • Hepatitis B • Hepatitis C • Alcohol	3 (43%) 3 (43%) 1 (14%)
AFP, median (range), at the time of transplant (µmol/L)	136.5 (11–593)
Tacrolimus trough level (ng/ml), median (range) trough level between transplant and recurrence	0–3 months post LT 11.05 (3.5–15.1) 4 months post LT -recurrence) 8.55 (2-9.7)
**Pathologic Factors**:	
Largest tumor diameter (cm), mean ± SD	4.6 ± 2.4
Multiplicity of lesions on the explanted liver (%): • 1 lesion • 2 lesions • 3 lesions • 4 lesions	3 (43%) 4 (57%) 0 (0%) 0 (0%)
Histologic grade: • Well-to-moderately differentiated • Moderately-differentiated • Poorly-differentiated • Unclassified	5 (72%) 1 (14%) 1 (14%) 0 (0%)
Presence of microvascular invasion on the explant HCC (%)	4 (57%)
Macrovascular invasion	1 (14%)
**HCC related outcome**:	
Vital status • Alive • Dead	1 (14%) 6 (86%)
Overall survival (median (range), in years	7 (1.5–13)
Time to recurrence (median (range), in months	33 (6–84)
Site of recurrence: • Liver • Lung • Spinal column	3 (43%) 3 (43%) 1 (14%)
Primary treatment for recurrence: • Liver resection • Lung resection • Radiotherapy	3 (43%) 3 (43%) 1 (14%)

### Nanostring nCounter tumor signaling 360 panel

Five µm thick scrolls were sectioned from formalin-fixed, paraffin-embedded (FFPE) tumor tissue blocks. Total RNA was extracted using the RNeasy FFPE Kit (QIAGEN) according to the manufacturer’s protocol, using xylene for deparaffinization. The Nanostring nCounter tumor signaling 360 panel (760 genes) was utilized for gene expression analysis, and data was acquired with Nanostring’s Digital Analyser (FOV, 555). To account for background noise and sample variation across multiple runs on the nCounter platform, raw gene expression count data was normalized in NanoString nSolver 4.0 using six positive controls and eight negative controls [14]. A manual calculation of the background threshold was carried out by taking the Mean of negative controls ± 2 standard deviations of negative control probes to exclude lowly expressed targets before conducting gene expression analysis. The nSolver 4.0 software integrated housekeeping gene probes and facilitated data normalization through the GeNorm Algorithm [14]. To address the issue of false discoveries, q-values were derived from the p-values obtained from NanoString nSolver analysis to control the false discovery rate (FDR) [15, 16]. A q-value cutoff of 0.05 was applied to identify differentially expressed genes (DEGs).

### Gene ontology and pathway enrichment analysis

Gene Ontology (GO) is a unique database that describes the characteristics and cell location of each gene [17]. Identifying GO terms elucidates how genes function by providing insight into their molecular activity, cellular actions, and their locations within the cells. WikiPathways is a database containing a large number of known genetic pathways of gene annotation [18]. The GO term enrichment and WikiPathways analyses, using DEGs, were performed in Enrichr [19]. This allowed for the identification of enriched biological processes and pathways with an adjusted p-value < 0.001, thus indicating significant differences.

### Protein-protein interaction (PPI) network analysis

STRING (version 11.5) [20], a search tool for retrieving interacting genes/proteins, was used to evaluate protein-PPI information. Homo sapiens was chosen as the organism of interest, with a minimum required interaction score set to medium confidence (0.4), while the remaining parameters were left at their default settings. The PPI interaction network generated (interactions.tsv file) was imported into the Cytoscape software (version 3.9.1) [21]. The application plugin of CytoHubba [22] was used for further analysis of the PPI network to identify the top 10 hub genes from the network based on the multiple correlation clustering (MCC) algorithms [23].

### Cibersortx analysis

Cibersortx [24] software was utilized to determine the differential abundance of immune cells between initially resected and recurrent tumors based on gene expression data. Cibersortx is an established bioinformatics algorithm that uses a matrix decomposition approach to infer the proportions of cell types in each tissue based on gene expression data [24]. The signature gene matrix used in the analysis contained expression counts of signature genes for 22 distinct human immune cells. The analysis involved extracting a submatrix from the gene expression count matrix and submitting it to the Cibersortx website (https://cibersortx.stanford.edu/) for analysis. Batch correction was enabled in B-mode, with the single-cell expression matrix collapsed into a bulk matrix as reference, for the NanoString nCounter dataset [25, 26].

### Deep learning-based analysis

#### Approach

In our deep learning (DL)-based approach, we leveraged prior biological knowledge obtained from pathways and GO term enrichment analyses to curate the list of pathways for analysis. Specifically, pathways with an adjusted p-value < 0.05 containing five or more differentially expressed genes, and GO terms with an adjusted p-value < 0.05 comprising ten or more differentially expressed genes, were included in our pathway-centric DL approach. Our objective was to identify subsets of genes within critical pathways whose expression levels could effectively distinguish primary from recurrent tumors. This process involved training independent supervised classifiers for each biological pathway and GO term.

Given the constraint of limited data, the risk of overfitting was a significant concern. To mitigate this challenge, we employed a two-step strategy. First, we augmented the dataset by adding gene expressions of primary tissue HCC samples from The Cancer Genome Atlas (TCGA) [27]. We excluded 8 samples of combined hepatocellular carcinoma and intrahepatic cholangiocarcinoma, 3 samples of fibrolamellar carcinoma and 2 samples of recurrent tissue type (liver cancer not otherwise specified), resulting in a final cohort of 360 HCC primary tissue samples. This augmentation, however, resulted in imbalanced class distributions with a greater number of primary tissue samples compared to recurrent ones. To address this imbalance, we trained supervised classifiers on pairs of tissues, where the learning objective was to differentiate between primary-primary and primary-recurrent tumor pairs.

#### Model architecture

The classifier model processed pairs of gene expression values within specified pathways or Gene Ontology (GO) terms, subsequently assigning a binary label to indicate whether the gene expression pairs corresponded to primary-primary tissue pairs or primary-recurrent sample pairs. The model architecture was meticulously designed to capture pairwise relationships between expression levels of the same gene across both samples. This network design included a first hidden layer with 16 convolution filters that convolved across the sample dimension to learn expression patterns across the two samples. The output of this layer was connected to two successive fully connected layers, with 32 and 16 filters, respectively followed by the output layer. All hidden layers, including the convolution layer, utilized Rectified Linear Unit activation [28], while the output layer used Softmax activation to facilitate binary prediction. The same model architecture was consistently applied across all relevant pathways and GO term models, with the input layer’s size determined by the number of differentially expressed genes within the pathway or GO term.

To optimize model performance, binary cross-entropy loss was minimized using Adagrad optimization [29]. To enhance robustness, each pathway-centric model underwent 10 iterations of bootstrapping, where the train-test sets were randomly partitioned.

#### Data normalization

Combining different datasets, such as the HCC TCGA dataset and our experimental data, posed a major challenge due to variations in technologies and tissue type. These differences hindered direct comparisons. To address this issue, we implemented a rigorous form of normalization, wherein the gene expression levels of each sample within each pathway were independently scaled to adhere to a standard normal distribution. As a result, our models were trained solely on the relative expression patterns among pathway genes, mitigating the impact of dataset-specific biases and ensuring the compatibility and comparability of the combined datasets.

#### Feature importance analysis

To identify crucial features, we computed SHAP (SHapley Additive exPlanations) values for each feature (gene) in each model using the test feature set. To ensure that only features consistently contributing to the model’s performance were considered, we set a threshold, requiring features to have a SHAP value of 0.01 or higher in at least 50% of the high-performing models across the bootstraps of the specific pathway or GO term. The SHAP value threshold of 0.01 was heuristically determined, and a model was deemed high-performing if it achieved greater than 80% classification accuracy on the test dataset. For instance, in a particular pathway, if 8 out of the 10 bootstrapped models achieved greater than 80% classification accuracy, only those features that had a SHAP value of 0.01 or higher in at least 4 of the 8 high-performing models were considered important.

## Results

### Functional enrichment analysis of DEGs on HCC recurrence after liver transplantation

**Fig. 1:**
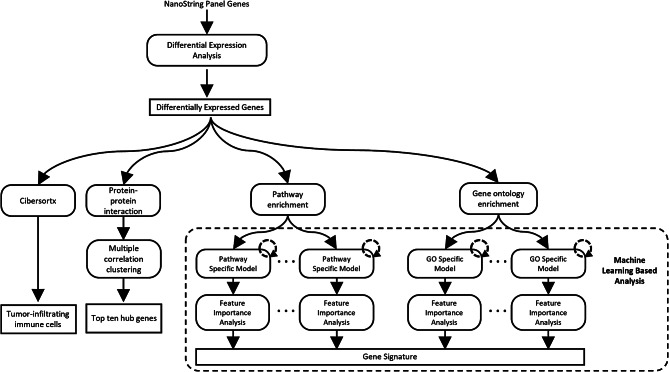
Analysis workflow of this study

**Fig. 2:**
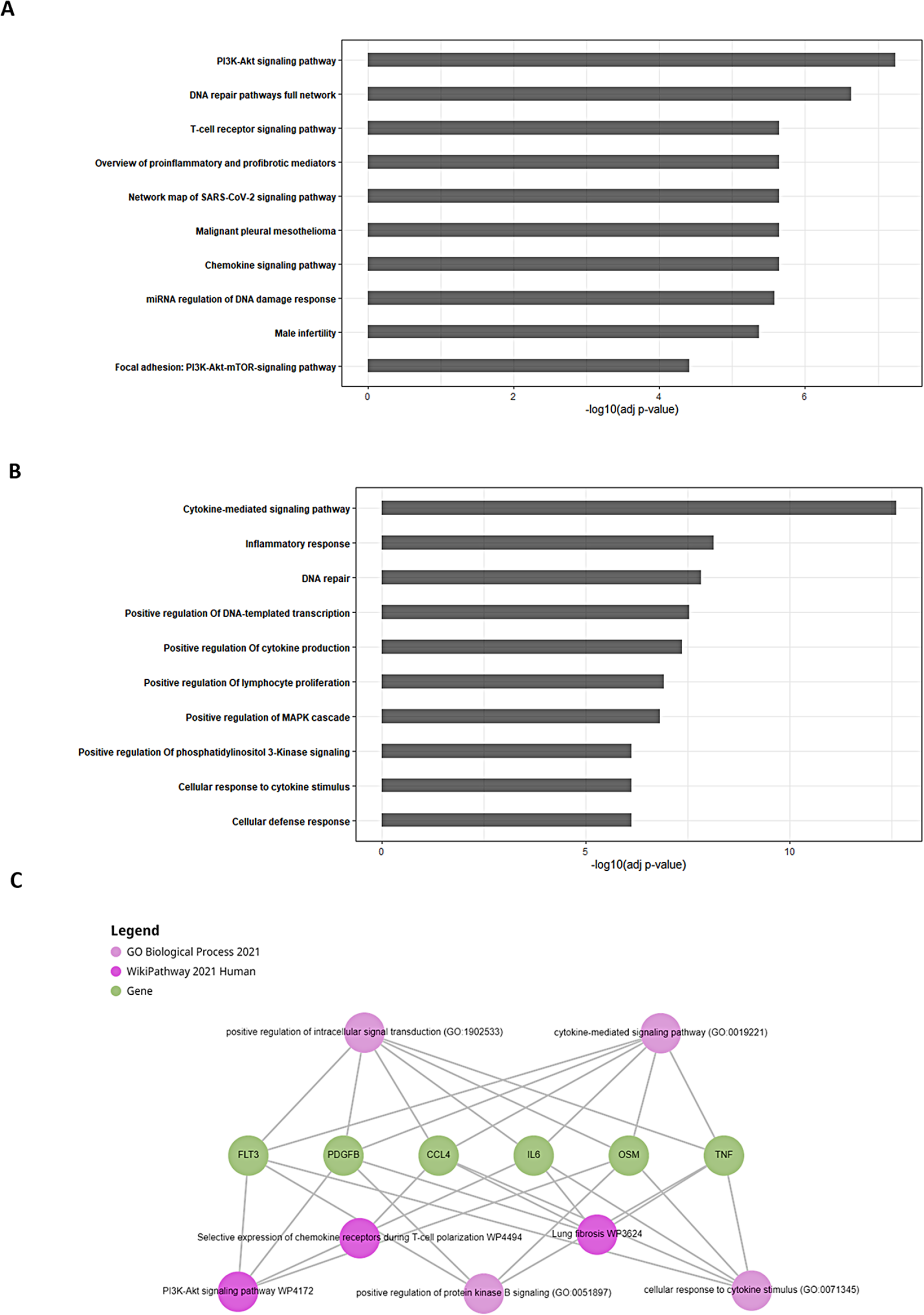
Functional enrichment analysis of the DEGs on HCC recurrence. (**A**) The top 10 pathway analysis results of the DEGs. (**B**) The top 10 Gene Ontology of biological process analysis results of the DEGs. (**C**) Network visualization of top 5 prominent enriched pathways and GO terms with the filtering of minimum of 5 links per gene

Paired tumor samples were collected from 7 patients at both the initial resection and recurrence to investigate the tumor genetic evolution of HCC recurrence. A q-value cutoff of 0.05 and | Fold change | ≥ 1.5 resulted in a total of 162 DEGs, with 160 upregulated and 2 downregulated genes. The heatmap of DEGs was presented (**Supplementary Fig. 1**). This data was further processed for different downstream analyses (Fig. 1). To investigate the function of the 162 DEGs regarding HCC recurrence during genetic evolution, pathways enrichment analysis and gene ontology (GO) assessment were performed. The top 10 most significantly enriched pathways were identified (Fig. 2A). PI3K-Akt signaling exhibited as the top pathway in the enrichment (WP4172). Other enriched pathways were identified with the association of cancer immunology and DNA damage response, such as DNA repair pathways full network (WP4946) and T-cell receptor signaling pathway (WP69). These enriched pathways potentially promoted HCC recurrence during disease progression. In the GO assessment of biological process, the GO term was primarily related to cytokine-mediated signaling pathway (GO:19221), inflammatory response (GO:6954) and positive regulation of cytokine production (GO:0001819) (Fig. 2B). The results indicated that the tumor microenvironment with cytokines mediated immunity significantly contributed to HCC recurrence. In the network constructed using the top 5 prominent enriched pathways and GO terms, each requiring a minimum of 5 links per gene, 6 DEGs (*FLT3, CCL4, IL6, OSM, TNF and PDGFB*) were identified as the most important bridge genes (Fig. 2C).

### Identification of top 10 hub genes in PPI network

**Fig. 3:**
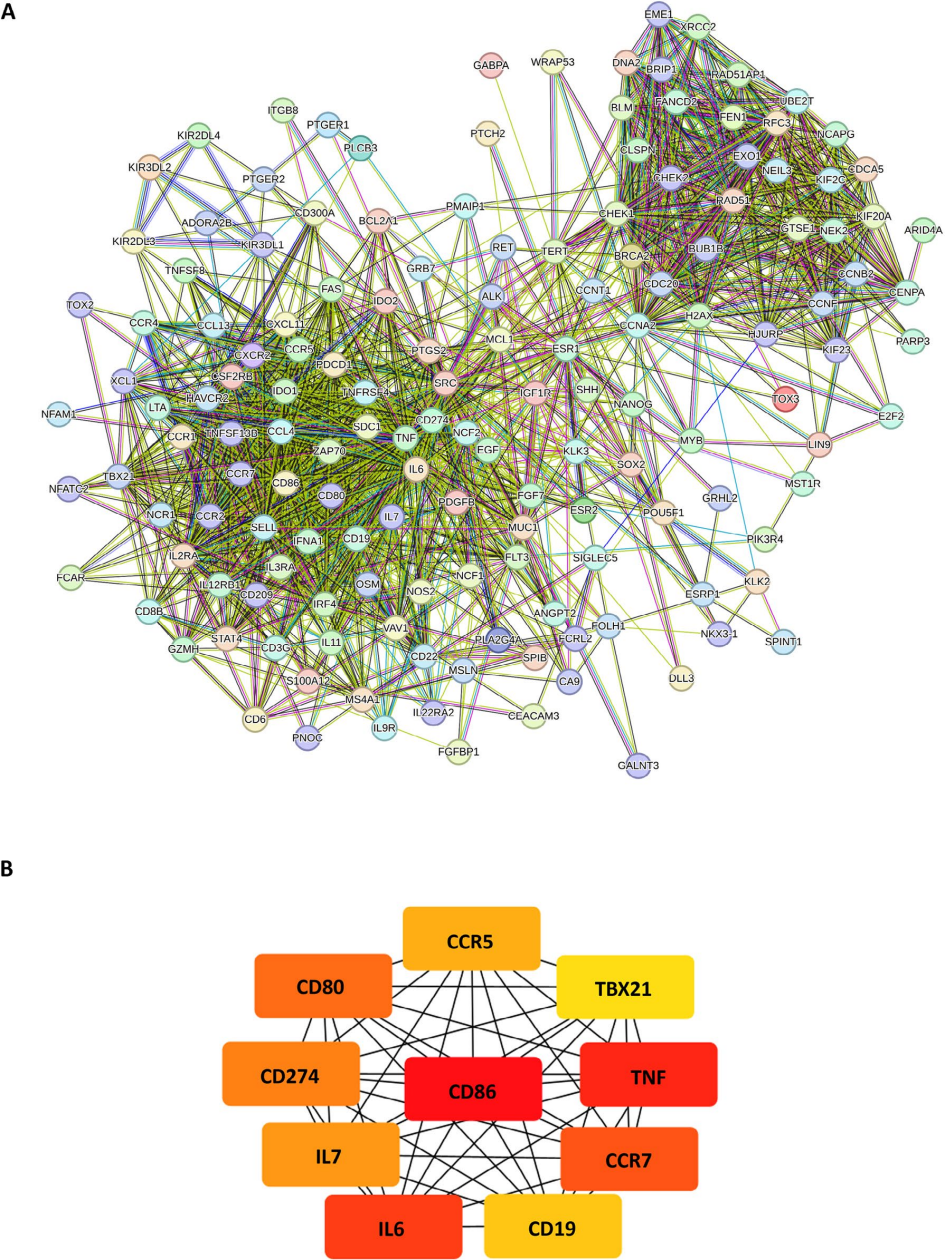
Protein-protein interaction (PPI) network analysis on HCC recurrence. (**A**) STRING analysis shows that DEGs are involved in known and predicted PPI. Network nodes represent proteins. Edges represent protein-protein associations. Different coloured lines represent different types of evidence used to predict associations. Red line: gene fusions; green line: gene neighbourhood; blue line: gene co-occurrence; purple line: experimentally determined; yellow line: text mining; light blue line: curated database; black line: co-expression and violet line: protein homology. (**B**) PPI network was subjected to Cytoscape using the CytoHubba MCC clustering algorithm to identify the top 10 hub genes in the HCC recurrent post-transplant tumors compared with the paired primary tumors. The 10 hub genes are displayed from red (high degree value) to yellow (low degree value)

The protein-protein interaction (PPI) network was constructed using the DEGs involved in the top 5 functional enrichment of pathways and GO terms. The genes were utilized as the seed to construct a medium confidence (0.4) PPI network on the STRING database [20]. Subsequently, the PPI network was subjected to Cytoscape using the CytoHubba MCC clustering algorithm to identify the top 10 hub genes. The PPI network for HCC recurrence post-transplant consisted of 160 nodes and 1555 edges (PPI enrichment p-value < 1.0 × 10 − 16) (Fig. 3A). In the MCC clustering analysis, the top 10 hub genes identified were CD86, TNF, IL6, CCR7, CD80, CD274, IL7, CCR5, CD19, and TBX21, ranked accordingly (Fig. 3B).

### **Cibersortx analysis of estimation of immune infiltration on HCC recurrence**

**Fig. 4:**
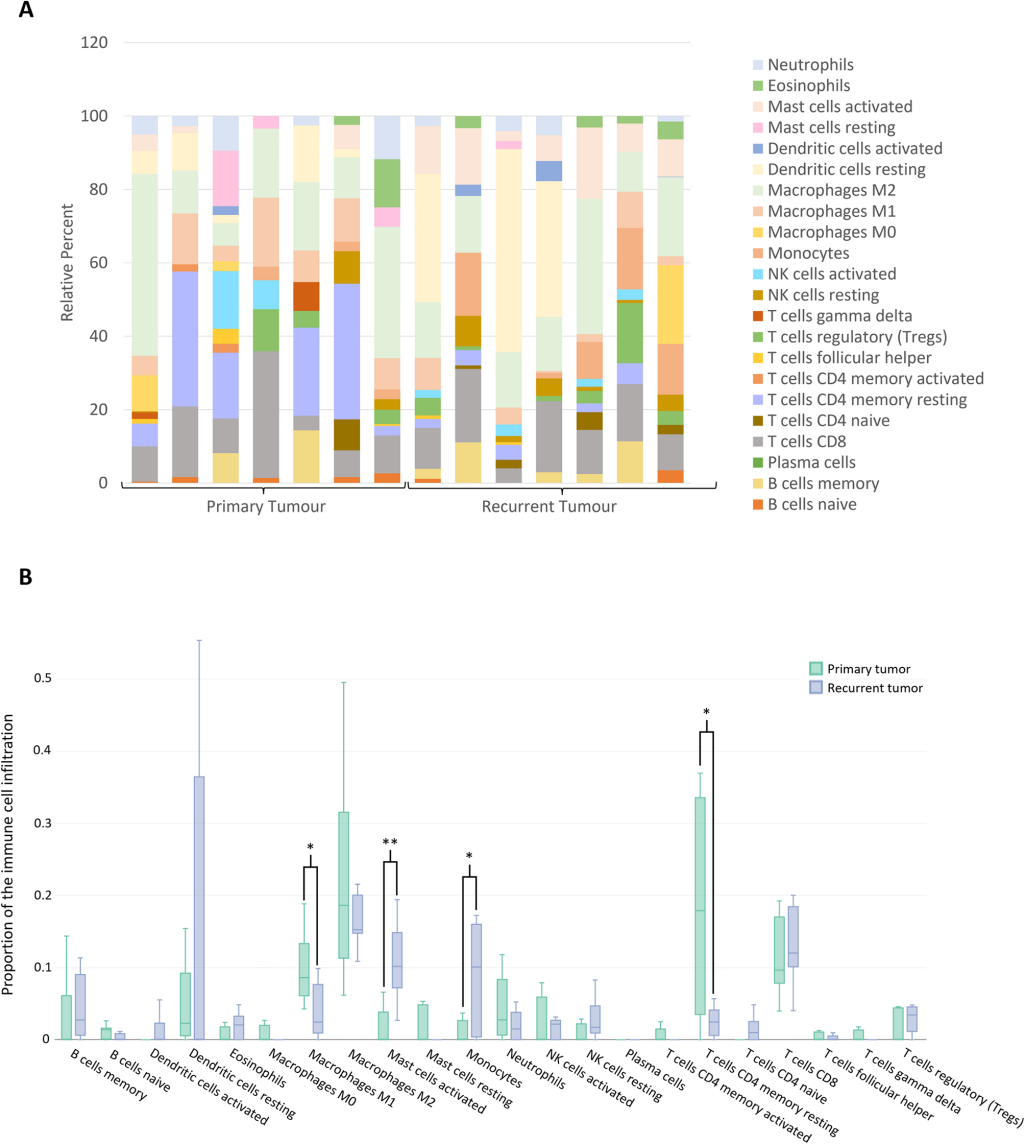
The landscape of immune infiltration in recurrent HCC samples. (**A**) Bar charts of 22 immune cell proportions in primary tumor and recurrent tumor samples. (**B**) Differential expression of different immune cell types between primary tumor and recurrent tumor samples. *, *p* ≤ 0.05; **, *p* ≤ 0.01; ***, *p* ≤ 0.001

Immune cell types and levels have been associated with cancer outcomes, with varying infiltration of immune cell types in the tumor microenvironment affecting the risk of cancer-free survival and overall survival [30–32]. In our functional enrichment analysis of recurrent HCC, cancer immunology played an important role in HCC recurrence after liver transplantation (Figs. 2 and 3). Therefore, Cibersortx analysis was performed to estimate immune cell infiltration based on gene expression profiles. The proportions of immune cells were determined in the paired tumor samples at both the initial resection and recurrence (Fig. 4A). The results showed decreased levels of resting CD4 + lymphocytes and M1 macrophages, while significantly increased infiltration of monocytes and activated mast cells (Fig. 4B).

### Deep learning-based approach to discern combinations of genes whose expression levels can discriminate between primary and recurrent tumors

**Fig. 5:**
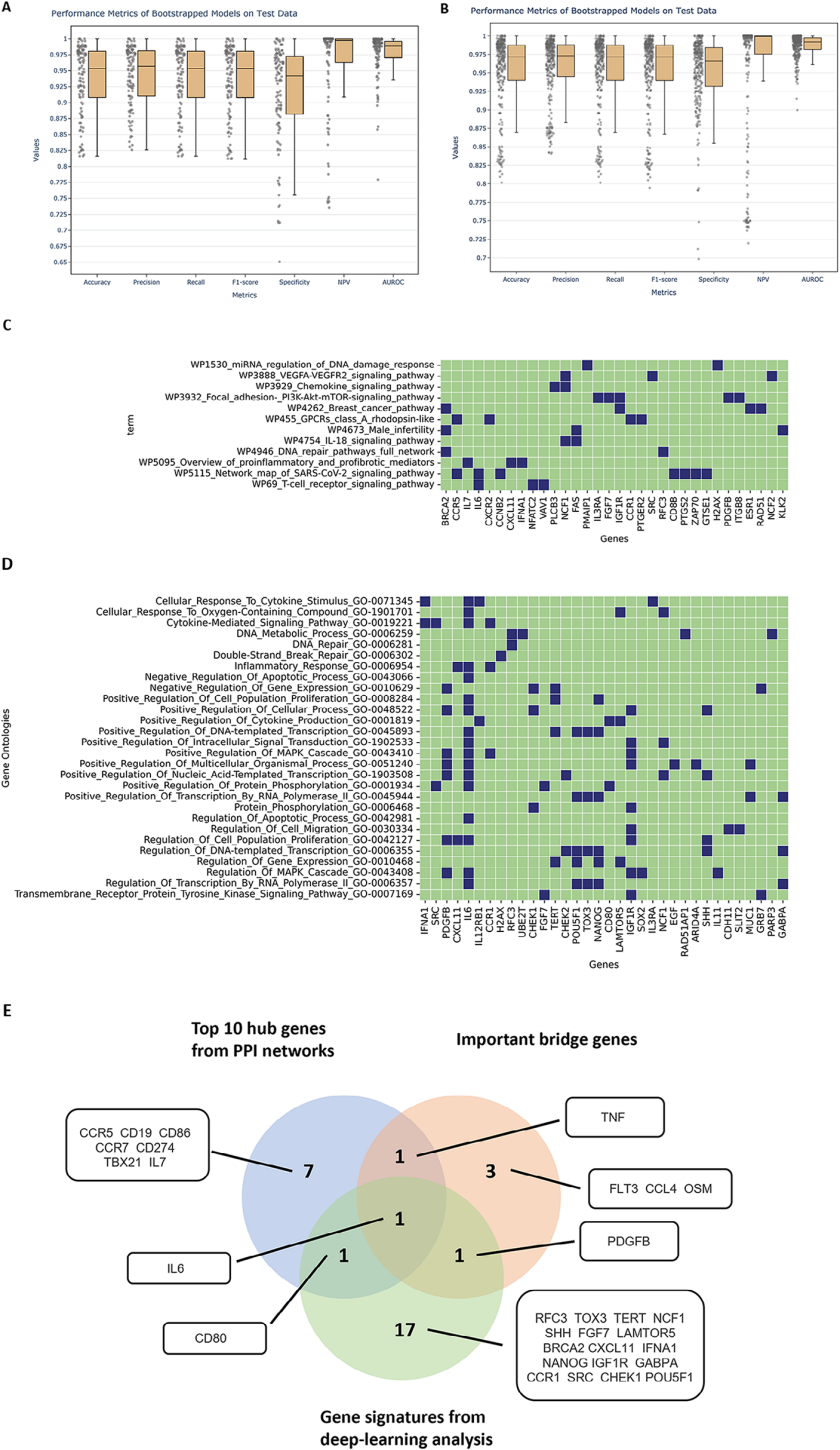
Identifying gene signatures of recurrent HCC tumors using pathway-centric modeling. (**A**) Performance metrics of bootstrapped pathway models on the test data, (**B**) Performance metrics of bootstrapped GO term models on the test data. Overall, both pathway and GO term-based models achieved good performance on test data with accuracy, precision, recall (sensitivity) metrics > 0.80. (**C**), (**D**) Genes in corresponding pathway and GO term that the models used to distinguish between primary and recurrent tissue. (**E**) Overlapping genes between gene signatures from deep learning-based analysis, important bridge genes and the top 10 hub genes from PPI networks

The selected models employed in the feature importance analysis exhibit strong overall performance (Fig. 5). The distribution of different performance metrics across bootstrapped samples for both the pathway and Gene Ontology (GO) models are illustrated in Fig. 5A and B. Specifically, the median specificity and sensitivity values on the test set for the biological pathway models were 0.964 and 0.918, respectively, while the median specificity and sensitivity values for the GO term models were 0.982 and 0.956, respectively.

It is noteworthy that, despite utilizing a relatively small training dataset, we effectively mitigated the risk of overfitting, as evidenced by the balanced sensitivities and specificities of the models. Within this set of high-performing models, we identified key genes based on their high SHAP values in at least 50% of their respective bootstrapped iterations. This stringent criterion ensures the identification of genes consistently utilized by the models to discern primary from recurrent cases. The ensuing analysis reveals key discriminating genes in association with enriched pathways and GO terms as shown in Fig. 5C and D. Importantly, PI3K/Akt signaling was consistently identified as the important pathway in recurrent HCC (Fig. 5C**)**. The deep-learning analysis revealed 28 Gene Ontology (GO) terms and 12 pathways featuring 34 and 32 genes, respectively, exhibiting differential expression in recurrent tissues compared to primary tissues. A total of 12 genes overlapped between GO term-centric and pathway-centric analysis. We computed a gene discriminant score based on the number of pathways and GO terms the gene appeared as a good discriminator between the two classes. The top ranked 20 genes based on the discriminant score is shown in Supplementary Fig. 2. Among them, *IL6* emerged as the topmost gene, showing the highest enrichment in the GO terms (Fig. 5D) and overlapping with both the important bridge genes and the top ten hub genes (Fig. 5E). Furthermore, we applied our 20-gene signatures to conduct a disease-free analysis for TCGA HCC dataset. Notably, patients with high expression levels of the 20-gene signatures (Altered group, z-score > 2) exhibited a significantly shorter median disease-free period compared to those with lower expression levels (Unaltered group), 15.70 months vs. 29.66 months (**Supplementary Fig. 3)**.

## Discussion

The PI3K/Akt signaling pathway played the most significant role in recurrent HCC post-transplant. Additionally, the enriched GO terms revealed that the DEGs primarily contributed to cytokine-mediated signaling in tumor microenvironment during HCC recurrence. We further investigated the immune cell infiltration in recurrent HCC during tumor evolution. All the recurrent tumors exhibited a significantly higher monocyte and lower M1 macrophage infiltration. Emerging evidence indicates that oncogenic signaling, such as CTNNB1, PI3K/PTEN/AKT/mTOR, p53, NF-κB, and RAS/RAF/MAPK signaling significantly regulates the tumor microenvironment by recruiting immunosuppressive cells and decreasing anti-tumor immune cells infiltration to promote tumor progression [33–35]. Oncogenic signaling can also lead to the upregulation of immune-escape proteins in cancers, such as CD274, CD80 and CD86 [36, 37]. Additionally, such signaling can promote the secretion of chemokines and cytokines, which can recruit immunosuppressive cells for tumor progression [33]. Our findings revealed that PI3K/Akt pathway was the main oncogenic signaling in HCC recurrence. PI3K/Akt pathway was suggested to coordinate the upregulation of cytokines, such as IL6 and IL7 in tumor microenvironment during HCC recurrence. Intervening upon the PI3K/Akt/mTOR pathway has also been the subject of therapeutic investigation in the clinical setting for transplant recipients with hepatocellular carcinoma; prospective evaluation of sirolimus demonstrated benefits in overall short-term post-transplant survival and recurrence-free survival compared to calcineurin inhibitor-based immunosuppression [38].

This cytokine secretion potentially recruited the activated mast cells and monocytes infiltration along with lower M1 macrophages to promote HCC recurrence. Specifically, the infiltration of mast cells has been reported in HCC progression through angiogenesis and tumor growth [39]. Monocytes can promote tumor angiogenesis, invasion and metastasis in HCC [40]. M1 macrophages are regarded as anti-tumorigenic through phagocytosis of cancer cells [41]. As a result, the effective macrophages exclusion contributed to the evaluation of aggressive biological behaviors in HCC recurrence.

Our deep learning analysis pinpointed 20 key genes as genetic signature for recurrent HCC, with *IL6* standing out as the most significant signature, overlapping with both the crucial bridge genes and the top ten hub genes. IL6 is one of the well-characterized pro-tumorigenic cytokines associated with tumour-associated inflammation and tumorigenesis in HCC [42, 43]. Furthermore, IL6 has been reported as a pivotal player in the activation of the PI3K/Akt/mTOR signaling and serves as a prognostic biomarker in HCC [44, 45]. IL6 also plays an important role in proliferation, migration, invasion, and malignant progression by activating the mTOR signaling in HCC [46].

Our deep learning approach adopts a pathway-centric perspective, integrating prior knowledge of systems-level biology through biological functions and GO terms, while also considering interdependencies among genes that dictate biological processes. Dysregulated pathways are indicative of disease onset or progression, and often implicated in cancer progression. Therefore, identifying patient-specific pathway dysregulation can inform personalized treatment strategies. In contrast, traditional statistical methods for differential expression analysis, such as fold change, typically treat features in isolation and may not capture the complex interdependencies between them. We chose convolution neural network (CNN) to learn relative differences rather than absolute expression thresholds across pairs of patients within groups of functionally related genes. This choice mitigates bias and effectively addresses challenges associated with normalizing variations arising from different measurement techniques and individual biological differences. Our method was designed to capture both similarities and dissimilarities between patient pairs (primary-primary and primary-recurrent), ensuring robustness against variations. By contrasting the similarities within primary-primary pairs and dissimilarities between primary-recurrent pairs, our models can effectively discern the unique signaling patterns and dysregulations characteristic of recurrent tissues. Consequently, when our analysis highlights IL6 as an important gene, as determined by SHAP analysis, it signifies that IL6 has played a significant role within the intricate set of features learned by the models, formed through non-linear combinations of multiple genes. Our methodology augments primary tissue data from TCGA, enhancing the diversity of patient samples and improving the generalizability. This approach allows using deep learning algorithms to discern the subtle distinctions even with limited samples. We generated 20-gene signatures for HCC recurrence. Among these signatures, IL6 [47, 48], IGF1R [48, 49], NANOG [50], NCF1 [5], POU5F1 [52], TERT [53], SHH [54], CCR1 [55], SRC [56] and CXCL11 [57] have been reported to have clinical associations with the risk of HCC recurrence. Future investigations into the clinical diagnostic and prognostic utility of these 20-gene signatures should be conducted.

Overall, our results provide valuable insights that PI3K/Akt signaling potentially upregulated tumor immune-escape genes and mediated cytokine secretion to affect immune cell infiltration pattern during the disease progression of HCC recurrence. Our study was constrained by a small sample size and the utilization of a NanoString panel with a limited gene coverage. Unlike Next Generation Sequencing (NGS), this approach restricted our ability to identify novel genes and pathways associated with HCC recurrence. To address this limitation, in the future, large-scale genome-wide association studies using NGS should be prioritized to further investigate HCC recurrence. Moreover, the understanding of how liver cancer modulates immune responses through tumor-intrinsic mechanisms remains limited. Further mechanistic research is needed to fully explore this potential and advance our knowledge of liver cancer immunology.

### Electronic supplementary material

Below is the link to the electronic supplementary material.

Supplementary Material 1

## Data Availability

GSE249913 is the accession number for our NanoString data.
