# Supplementary material for: Deep learning-based pathway-centric approach to characterize recurrent hepatocellular carcinoma after liver transplantation
Source: Hum Genomics. 2024 Jun 5;18:58. doi: 10.1186/s40246-024-00624-6 (PMC11151487; doi:10.1186/s40246-024-00624-6)
Supplement: Supplementary file 1 — Supplementary Material 1 [file 40246_2024_624_MOESM1_ESM.pdf]

Supplementary Fig. 1

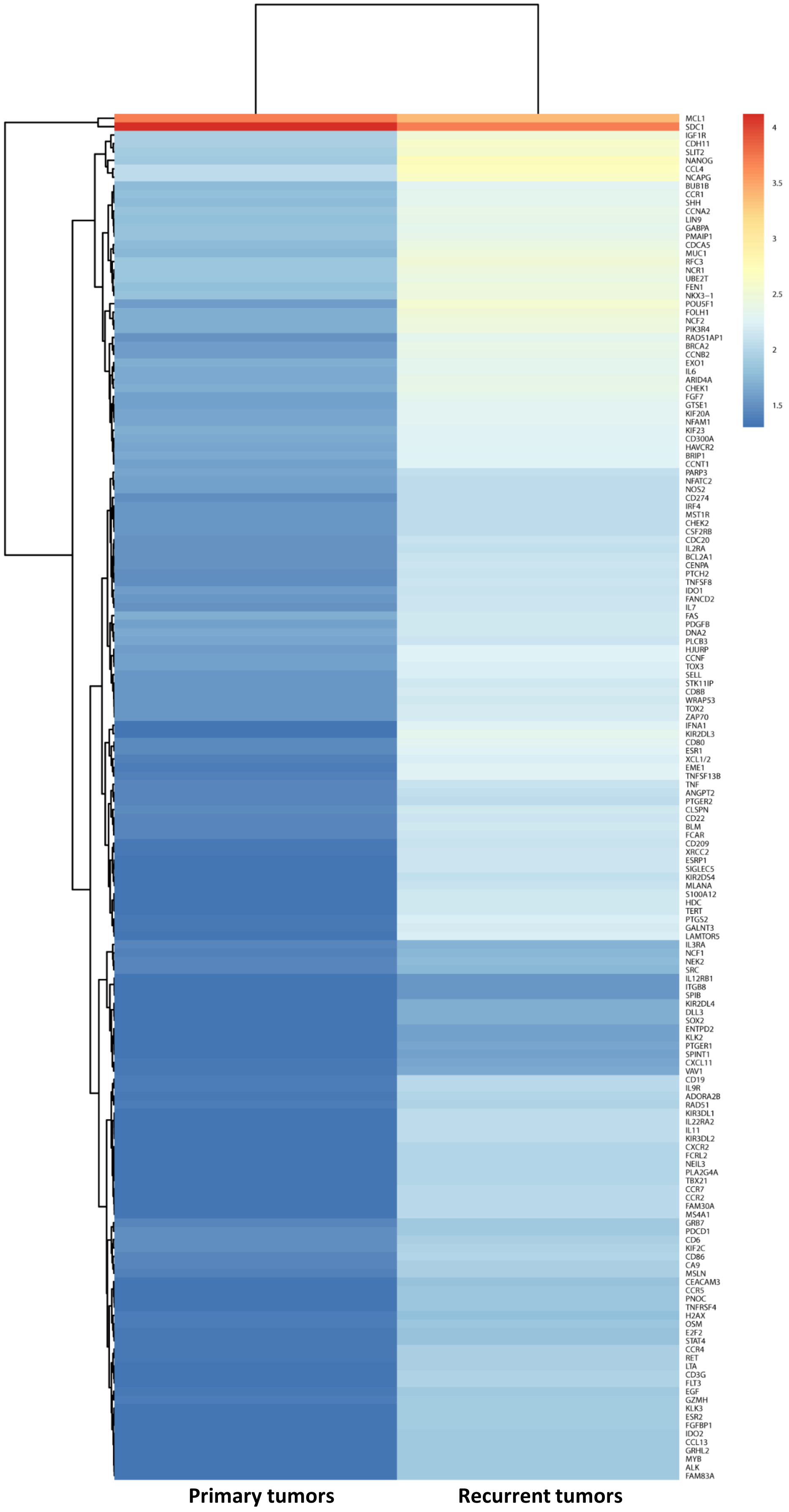

Supplementary Figure 1. Differentially expressed genes (DEGs). The heatmap shows the 162 DEGs of HCC recurrent post-transplant tumors compared with the paired primary tumors (q-value ≤ 0.05 and|Fold change| ≥ 1.5).

Supplementary Fig. 2

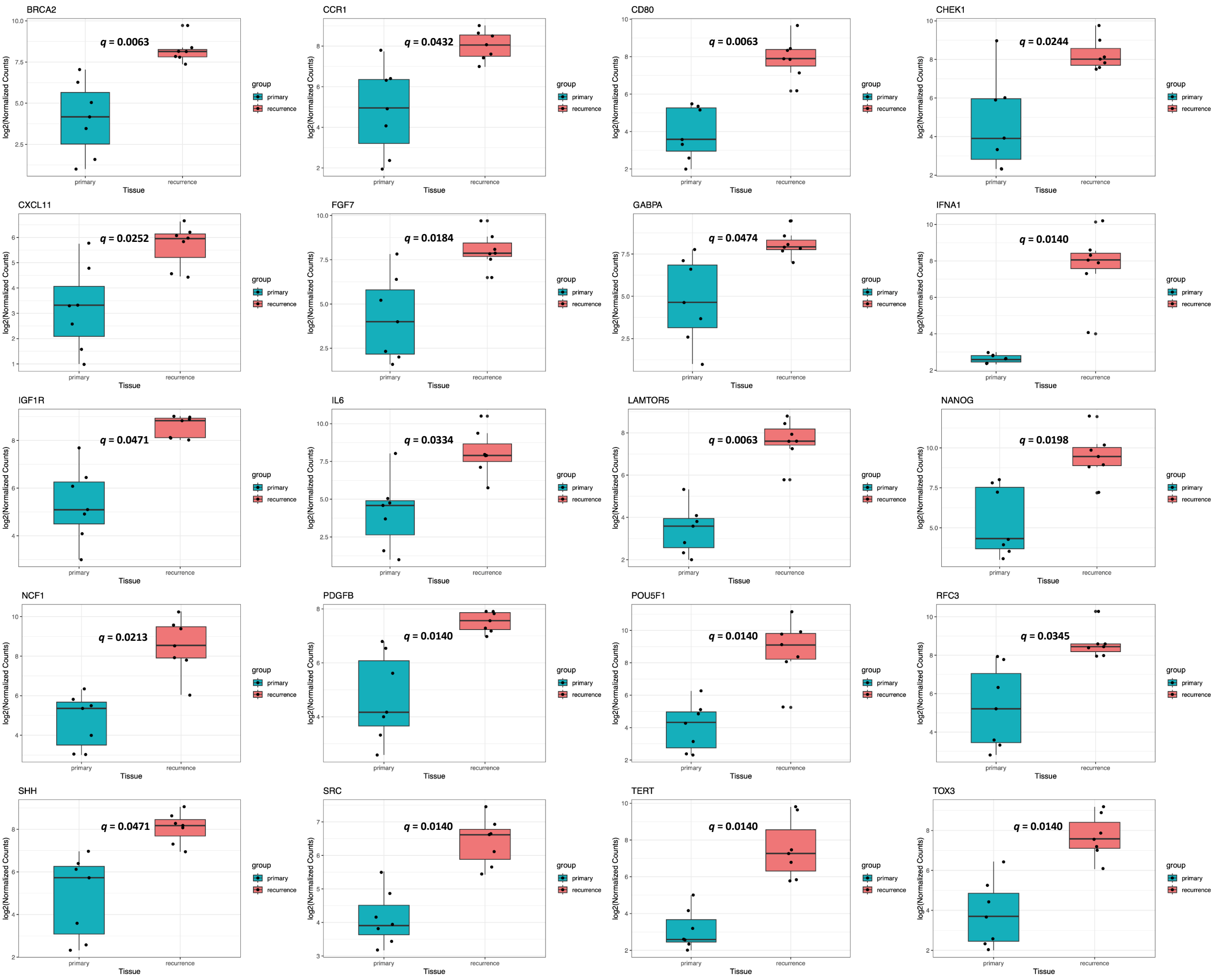

Supplementary Figure 2. Identification of 20 genes for recurrent HCC post-transplant in a pathway-centric modeling machine learning-based analysis.

Supplementary Fig. 3

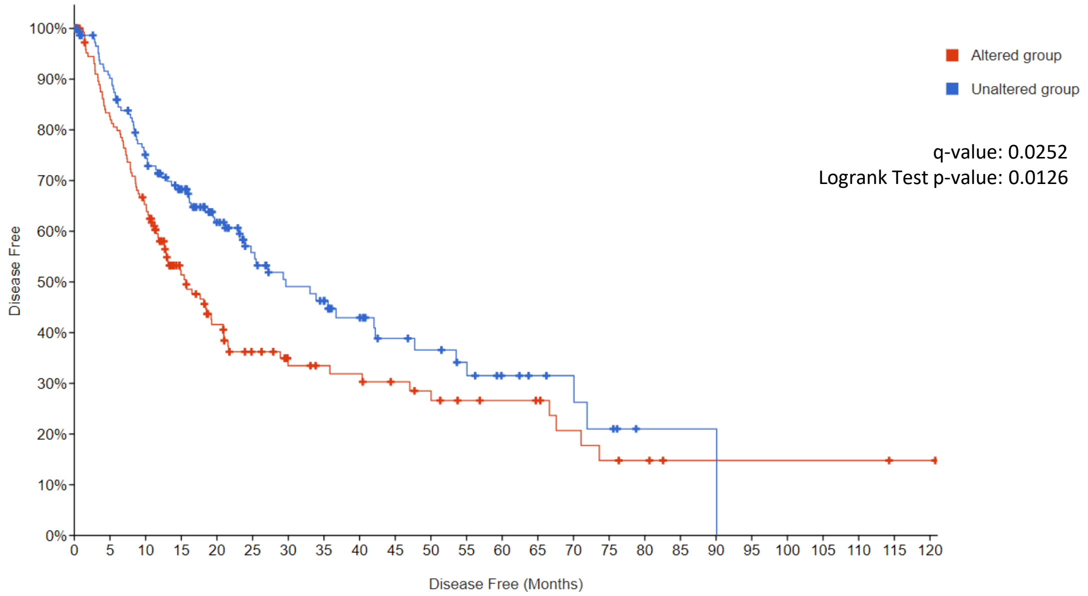

Number at risk (n)

Altered group	157	119	93	55	40	30	23	21	20	17	15	12	11	10	7	5	4	2	2	2	2	2	2	1	1
Unaltered group	151	128	101	83	59	44	35	32	24	18	16	13	9	7	6	4	1	1	1	0	0	0	0	0	0

Survival plot summary	Number of cases, Total	Number of events	Median months disease free (95% CI)
Altered group	157	93	15.70 (12.61 - 20.93)
Unaltered group	151	73	29.66 (23.95 - 47.73)

Supplementary Figure 3. Association between upregulation of 20-gene signatures and reduced disease-free survival in HCC patients.
